# Nasal polyposis in pediatric patients with Cornelia de Lange syndrome: endoscopic diagnosis, treatment and follow up in two case reports

**DOI:** 10.1186/s13052-023-01454-3

**Published:** 2023-07-16

**Authors:** Roberta Onesimo, Rita De Santis, Chiara Leoni, Mario Rigante, Marco Piastra, Elisabetta Sforza, Angelo Selicorni, Giuseppe Zampino

**Affiliations:** 1 Rare Diseases Unit, Fondazione Policlinico Universitario Agostino Gemelli–IRCCS, Largo Gemelli 8, 00168 Rome, Italy; 2 Pediatric Unit, Fondazione Policlinico Universitario Agostino Gemelli–IRCCS, Rome, Italy; 3 Otorhinolaryngology Unit – Fondazione Policlinico, Universitario Agostino Gemelli–IRCCS, Rome, Italy; 4 Pediatric Intensive Care Unit, Fondazione Policlinico Universitario Agostino Gemelli -IRCCS, Rome, Italy; 5 Pediatric Unit, ASST Lariana, Como, Italy; 6 Università Cattolica del Sacro Cuore, Rome, Italy

**Keywords:** Cornelia de Lange Syndrome, Nasal polyps, Obstructive sleep apnea, Nasal obstruction, Endoscopic surgery, Case report

## Abstract

**Background:**

Cornelia de Lange syndrome is a rare genetic disease with otolaryngological involvement. The classic phenotype is characterized by distinctive facial features, intellectual disability, growth delay, hirsutism, and upper-limb reduction. Nasal polyposis was previously reported in association with chronic rhinosinusitis, however data about prevalence, diagnosis, treatment and prognosis are lacking for this cohort of patients, affected by rare disease.

**Case presentation:**

We describe the whole diagnostic and therapeutic workflow of nasal polyps in two pediatric patients with Cornelia de Lange, successfully diagnosed and treated by nasal endoscopy.

**Conclusion:**

Our report confirm that nasal endoscopy is a safe and useful tool in the diagnosis, treatment and follow-up of nasal polyps, even in Cornelia de Lange syndrome pediatric patients. We want to increase the alert for the detection of nasal polyps in patients with Cornelia de Lange syndrome since pediatric age. We recommend endoscopy in all patients with Cornelia de Lange syndrome and symptoms of chronic nasal obstruction and/or OSAS. Multidisciplinary team and sedation service could be useful in the management of Cornelia de Lange syndrome patients with airway obstruction symptoms and sleep disturbance when severe intellectual disability, autism or psychiatric findings are present.

**Supplementary Information:**

The online version contains supplementary material available at 10.1186/s13052-023-01454-3.

## Background

Cornelia de Lange syndrome (CdLS) is a rare genetic disease with a prevalence range from 1:10.000 to 1:40.000. An international consensus statement has defined cardinal features to define classic and non-classic CdLS [1]. The classic phenotype is characterized by distinctive facial features, intellectual disability, growth delay, hirsutism, and upper-limb reduction.

The diagnosis of CdLS is established in a proband with suggestive clinical features and/or by identification of pathogenic variant in NIPBL, RAD21, SMC3, BRD4, HDAC8 or SMC1A by molecular genetic testing [1, 2].

Many authors described maxillofacial and otolaryngological involvement in CdLS [3].

Sensorineural hearing loss is described in 80% of children with CdLS, with 40% being profoundly affected [4]. Other otolaryngological diseases are obstructive sleep apnea, laryngomalacia and rhinosinusitis.

Nasal polyps (NP) in CdLS [5, 6] were previously reported in association with chronic rhinosinusitis (CRS), however data about NP prevalence, diagnosis, treatment and prognosis are lacking for this cohort of patients.

We describe the whole diagnostic and therapeutic workflow of (NP) in two pediatric patients with CdLS, follow up information are reported, too.

## Cases presentation

### Case 1

This female patient come to our Center of Rare Diseases when she was 5. She was classified as a classic CdLS [1], NIPBL gene mutation confirmed the diagnosis.

She have had severe gastro esophageal reflux disease (GERD), surgical treatment (Nissen fundoplication) was required. She presented severe obstructive sleep apnea syndrome (OSAS) that was treated by Continuous Positive Airway Pressure (CPAP). She did not have a history of CRS or allergy.

When she was 12, parents reported persistent nasal obstruction and worsening of sleep disorder.

Endoscopy revealed a nasal polyp (Video 1, supplementary materials) in the middle meatus, protruding in the right nasal fossa with mild nasal obstruction (endoscopic Type III nasal polyposis according to Stammberger Clinical classification) [7]. The NP diagnosis was histological proved.

The patient underwent intranasal steroid therapy for 6 months, with sleep disturbance improvement. After 2 years, endoscopy revealed absence of nasal polyposis.

### Case 2

This female patient was diagnosed in the first year of age with CdLS, classic form [1].

She developed severe intellectual disability and moderate GERD treated by proton pump inhibitor. Recurrent nasal obstruction was detected, and OSAS was diagnosed when she was 6, CPAP treatment was performed.

When she was 17, because of worsening of nasal obstruction symptoms, endoscopy was performed and revealed NP associated with CRS.

Multiple unilateral polyps were found, one in the nasal fossa and a second one in the maxillary sinus, as confirmed by CT scan (Type II according to Stammberger classification), the patient was successfully treated by endoscopic surgery.

Endoscopic recurrence of NP occurred after one year, nasal steroid therapy lead to resolution, as confirmed by endoscopy.

## Discussion and conclusion

CdLS is a rare genetic syndrome with a high involvement of maxillofacial manifestations. No specific treatment is available, but multidisciplinary approach can improve the quality of life of patients [8].

NP is a rare event in healthy children; it is usually bilateral, commonly associated with asthma [9]. Most studies about children with CRS shown a lower prevalence of NP when compared to adults because there are differences in the inflammatory response [10].

NP was occasionally described in adolescents and adults with CdLS [5, 8], despite this, chronic nasal obstruction and sleep disturbance are commonly reported [3], we suppose that NP prevalence can be underestimate in this cohort of patients due to lack of compliance, intellectual disability and autism.

In our experience (case 1) NP can be diagnosed even in patients with CdLS without CRS. We hypothesized that in some CdLS patients NP could be the result of chronic inflammation due to GERD, craniofacial features, velar insufficiency and generalized hypothonia. A long-term acid exposure could justify an increase in the prevalence of airway chronic inflammation in CdLS compared to the general population, especially in pediatric age.

Our report confirm that nasal endoscopy is a safe and useful tool in the diagnosis, treatment and follow-up of NP, even in CdLS pediatric patients.

We want to increase the alert for the detection of NP in patients with CdLS since pediatric age. We recommend endoscopy in all patients with CdLS and symptoms of chronic nasal obstruction and/or OSAS. Multidisciplinary team (otolaryngology, maxillofacial surgeon, pediatrician with expertice in disability, neurologist) and sedation service could be useful in the management of CdLS patients with airway obstruction symptoms and sleep disturbance when severe intellectual disability, autism or psychiatric findings are present.

## Electronic supplementary material

Below is the link to the electronic supplementary material.

Supplementary Material 1

Supplementary Material 2

## Data Availability

The data that support the findings of this study are available on request from the corresponding author, [RO]

## Abbreviations

CdLS: Cornelia de Lange syndrome
CRS: chronic rhinosinusitis
NP: nasal polyps
GERD: gastro esophageal reflux disease
OSAS: obstructive sleep apnea syndrome
CPAP: Continuous Positive Airway Pressure
