# Supplementary material for: Nasal polyposis in pediatric patients with Cornelia de Lange syndrome: endoscopic diagnosis, treatment and follow up in two case reports
Source: Ital J Pediatr. 2023 Jul 16;49:85. doi: 10.1186/s13052-023-01454-3 (PMC10350250; doi:10.1186/s13052-023-01454-3)
Supplement: Supplementary file 1 — Supplementary Material 1 [file 13052_2023_1454_MOESM1_ESM.pdf]

CARE Checklist of information to include when writing a case report

CARE case report guidelines

(cc) BY-NC-ND

Topic	Item	Checklist item description	Reported on Line
**Title**	1	The diagnosis or intervention of primary focus followed by the words "case report"	1-2
**Key Words**	2	2 to 5 key words that identify diagnoses or interventions in this case report, including "case report"	47-48
**Abstract (no references)**	3a	Introduction: What is unique about this case and what does it add to the scientific literature?	30-46
	3b	Main symptoms and/or important clinical findings	37-38
	3c	The main diagnoses, therapeutic interventions, and outcomes	37-38
	3d	Conclusion—What is the main "take-away" lesson(s) from this case?	39-45
**Introduction**	4	One or two paragraphs summarizing why this case is unique (**may include** references)	50-58
**Patient Information**	5a	De-identified patient specific information	66-79
	5b	Primary concerns and symptoms of the patient	70-82
	5c	Medical, family, and psycho-social history including relevant genetic information	67-71; 80-84
	5d	Relevant past interventions with outcomes	67-75; 80-87
**Clinical Findings**	6	Describe significant physical examination (PE) and important clinical findings	74; 88
**Timeline**	7	Historical and current information from this episode of care organized as a timeline	67-71; 80-84
**Diagnostic Assessment**	8a	Diagnostic testing (such as PE, laboratory testing, imaging, surveys).	73-75; 85-87
	8b	Diagnostic challenges (such as access to testing, financial, or cultural)	na
	8c	Diagnosis (including other diagnoses considered)	74; 84
	8d	Prognosis (such as staging in oncology) where applicable	na
**Therapeutic Intervention**	9a	Types of therapeutic intervention (such as pharmacologic, surgical, preventive, self-care)	76; 87
	9b	Administration of therapeutic intervention (such as dosage, strength, duration)	na
	9c	Changes in therapeutic intervention (with rationale)	na
**Follow-up and Outcomes**	10a	Clinician and patient-assessed outcomes (if available)	76; 86
	10b	Important follow-up diagnostic and other test results	76-77
	10c	Intervention adherence and tolerability (How was this assessed?)	76-77; 88-89
	10d	Adverse and unanticipated events	na
**Discussion**	11a	A scientific discussion of the strengths AND limitations associated with this case report	92-104
	11b	Discussion of the relevant medical literature **with references**	92-104
	11c	The scientific rationale for any conclusions (including assessment of possible causes)	105-112
	11d	The primary "take-away" lessons of this case report (without references) in a one paragraph conclusion	105-112
**Patient Perspective**	12	The patient should share their perspective in one to two paragraphs on the treatment(s) they received	na
**Informed Consent**	13	Did the patient give informed consent? Please provide if requested	Yes ☐ Nox☐
